# Human Tongue Thermography Could Be a Prognostic Tool for Prescreening the Type II Diabetes Mellitus

**DOI:** 10.1155/2020/3186208

**Published:** 2020-01-14

**Authors:** Usharani Thirunavukkarasu, Snekhalatha Umapathy, Palani Thanaraj Krishnan, Kumar Janardanan

**Affiliations:** ^1^Department of Biomedical Engineering, SRM Institute of Science and Technology, Kattankulathur 603203, Tamil Nadu, India; ^2^Department of Electronics & Instrumentation Engineering, St. Joseph's College of Engineering, Chennai 600119, Tamil Nadu, India; ^3^Department of General Medicine, SRM Hospital & Research Centre, Kattankulathur 603203, Tamil Nadu, India

## Abstract

Diabetes mellitus is one of the life threatening diseases over the globe, and an early prediction of diabetes is of utmost importance in this current scenario. International Diabetes Federation (IDF) reported nearly half of the world's population was undiagnosed and unaware of being developed into diabetes. In 2017, around 84 million individuals were living with diabetes, and it might increase to 156 million by the end of 2045 stated by IDF. Generally, the diagnosis of diabetes relies on the biochemical method that may cause uneasiness and probability of infections to the subjects. To overcome such difficulties, a noninvasive method is much needed around the globe for primary screening. A change in body temperature is an indication of various diseases. Infrared thermal imaging is relatively a novel technique for skin temperature measurement and turned out to be well known in the medical field due to being noninvasive, risk-free, and repeatable. According to traditional Chinese medicine, the human tongue is a sensitive mirror that reflects the body's pathophysiological condition. So, we have (i) analysed and classified diabetes based on thermal variations at human tongue, (ii) segmented the hot spot regions from tongue thermogram by RGB (red, green, blue) based color histogram image segmentation method and extracted the features using gray level co-occurrence matrix algorithm, (iii) classified normal and diabetes using various machine learning algorithms, and (iv) developed computer aided diagnostic system to classify diabetes mellitus. The baseline measurements and tongue thermograms were obtained from 140 subjects. The measured tongue surface temperature of the diabetic group was found to be greater than normal. The statistical correlation between the HbA_1c_ and the thermal distribution in the tongue region was found to be *r*^2^ = 0.5688. The Convolutional Neural Network has outperformed the other classifiers with 94.28% accuracy rate. Thus, tongue thermograms could be used as a preliminary screening approach for diabetes prognosis.

## 1. Introduction

Diabetes mellitus (DM) is an epidemic life threatening disorder characterized by elevated levels of blood glucose. The International Diabetes Federation (IDF) predicted that around one-fifth of the total population with diabetes in the world is from South East Asian countries. It was found that there are almost 84 million individuals suffering from DM in the year 2017. These figures were anticipated to increase to 156 million (86%) by the year 2045. Also IDF reported 425 million adults aged 20–64 years in 2017 are living with diabetes worldwide. By 2045, it is predicted to increase to about 629 million in adults aged 20–64 years [1]. Moreover, the World Health Organisation (WHO) estimated that in 2012, deaths caused by DM were found to be 1.5 million [2]. The globalized diabetes prevalence rate was estimated as 9% among men and 7.9% among women in 2014, increasing from 4.3% and 5.0% in 1980 due to population growth and ageing factors [3]. Prolonged asymptomatic phase of type 2 diabetes leads to micro- and macrovascular complications [4]. The researchers estimated about 45.8% or 174.8 million adults with diabetes are undiagnosed globally in the year 2013, and it is found to be 42.2 million adults in India [5]. The factors such as obesity, decreased physical activities, growing population, ageing, sedentary lifestyle lead to the rapid increase in diabetes prevalence rate in men and women [6]. Some study postulated that a hereditary factor can partly drive the risk of diabetes [7]. Various research studies observed a firm relationship between body mass index (BMI) and diabetes [8–13].

Generally, DM is categorized as type I and type II whereas type I DM is the degeneration of beta cells within the pancreas and type II DM is found when our body is unable to use the released insulin properly [14, 15]. The American Diabetes Association (ADA) reported that DM could be diagnosed based on HbA_1c_ criteria, either the fasting glucose or 2 h plasma glucose after a 75 g oral glucose tolerance test (OGTT) [16]. Several research studies suggested HbA_1c_ diagnostic criteria as the standard method for long term glycaemic control in diabetic patients [17, 18]. The biochemical analysis and glucometers are used as diagnostic methods to measure the glucose level for diabetic adults. These methods are producing precise results only after the damage had affected the nerves, blood vessels, and tissues. So, this inconvenience to the diabetic adults can be changed only by an alternative method for diagnosing DM [19]. The skin surface temperature was highly influenced by pathological and physiological changes in the human body [20]. Thermography is used to measure and analyse the distribution of thermal emission from the surface of the body [21]. Infrared thermal imaging is a noncontact, nonradiative, and risk-free detection tool [22]. The application of thermal imaging is persistently developing along with the technical advancements. Thermal imaging is used to evaluate dry eye [23], lower and upper extremities [24, 25], dermatology [26], Raynaud's disease [27, 28], diabetic foot ulcer [29], stress [30], and atherosclerosis [31].

Tongue diagnosis is a noncontact method to evaluate the condition of the patient's internal organs. The tongue is a sensitive mirror that reflects the pathophysiological condition of the body. According to East Asian Medicine, the internal organs such as liver, stomach, lung, heart, and urinary bladder meridians were directly connected to the corresponding reflex zones of the tongue [32]. Hu et al. have observed that tongue features are strongly correlated with the alanine aminotransferase (ALT) and aspartate aminotransferase (AST) which are important biomarkers for liver diseases [33]. Nakamura et al. have found a mass of neurofibroma in the tongue which indicates von Recklinghausen disease, which is an autosomal dominant neurogenetic disorder [34]. Jian et al. have evaluated the association between purple bluish tongue and increased platelet counts. Furthermore, they examined their relations with the recurrence of epithelial ovarian cancer [35]. Han et al. have found a distinctive microbiome coating on the tongue between colorectal cancer and healthy subjects [36]. Pang et al. have obtained promising results for appendicitis diagnosis based on the pathological changes on the tongue surface [37]. The association between blood perfusion rate and tongue thermograms for anaemic subjects was investigated by Xie and Zhang [38]. Liao et al. have identified that certain tongue features (tongue color, fur, and thickness) were related to the progression of diabetes with pyrogenic liver abscess (PLA) [39]. Zhihao et al. have found an unusual increase in tongue thermograms on coronary artery disease subjects compared with normal subjects due to the irregular metabolic rate [40]. Baek et al. have found that tongue surface temperature measured using infrared thermography could be the partial indicator of cold heat pathological patterns [41].

The proposed study was aimed (i) to analyse the thermal distributions in the tongue region between the normal and type II DM, (ii) to segment the hot spot regions (red component) using RGB based color histogram image segmentation method from tongue thermogram and extract the statistical features using gray level co-occurrence matrix (GLCM) algorithm, (iii) to classify the normal and type II DM based on the tongue surface temperature measurements and extracted features from the tongue region using various machine learning algorithms, and (iv) to develop the computer aided diagnostic system for the classification of type II DM.

## 2. Materials and Methods

### 2.1. Study Population

The tongue thermograms were collected from a free screening diabetic camp organized at SRM Hospital and Research Centre, Kattankulathur, Tamil Nadu, India with the approval of an institutional ethical committee with an ethical clearance number: 834/IEC/2015. The informed consent forms and questionnaires were obtained from all the subjects (*n* = 200). After scrutinizing the patient information sheet filled up by the participants, cases like fever, cardiovascular complications, arthritis, and renal failure were excluded from the study. Out of the 200 subjects who participated, a total of 140 subjects (Normal, *n* = 70 and type II DM, *n* = 70) aged between 20–50 years were included for the proposed study. The exclusion criteria involved for this proposed study were pregnant and nursing women and subjects with renal failures, thyroid problems, anaemia, and uncontrolled blood pressure; based on these criteria 60 subjects were excluded. The overall mean ± standard deviation of age for male subjects (*n* = 63) was found to be 44.60 ± 9.19 years and that of female subjects (*n* = 77) was 46.48 ± 9.45 years. Based on the World Health Organization (WHO) criteria, the standard HbA_1c_ value ≥6.5% was diagnosed as type II DM [42]. According to this criterion, the subjects were classified into two groups and tabulated in Table 1.

### 2.2. Baseline Measurements

The parameters such as height (cm), weight (Kg), body mass index (Kg/m^2^), systolic blood pressure (mmHg), hip circumference (cm), diastolic blood pressure (mmHg), waist circumference (cm), and Spo_2_ (%) were measured using a standard method. The biochemical variables such as the fasting blood glucose (FBG) (mg/dL), postprandial blood glucose (PPBG) (mg/dL), HbA_1c_ (%), and estimated average glucose (eAG) (mg/dL) were measured from the subjects blood sample using gold standard method [43, 44].

### 2.3. Measurement Protocol

**Figure 1:**
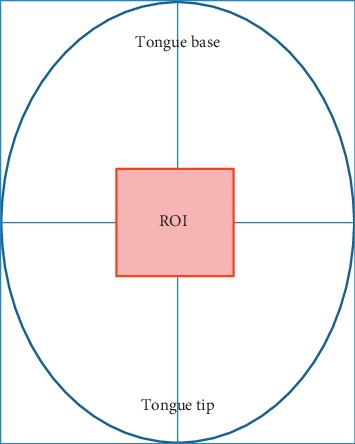
Template for positioning the region of interest (ROI) in tongue thermogram.

The tongue thermogram was acquired from the subjects using an infrared camera (FLIR A305 SC, FLIR Systems, USA). The subjects were instructed to remove all the metallic accessories and adapted to the controlled environment of 22–23°C and relative humidity of 50% [45]. The subjects were asked to be in a sitting position for ten minutes before the image acquisition procedure. The images were captured during the fasting condition for the total population studied. During the measurements, the subjects were advised to place their chin on a resting tool with widely opened mouth and to extend their tongue out with the tip pointing downward prior to the image acquisition process. The subjects were informed to close their mouth for two minutes as the prolonged extension might influence the blood perfusion as well as the temperature on the surface of the tongue. The images were captured at a distance of 0.3 meters from the tongue region of the subjects using a thermal camera. The tongue thermograms were analysed using thermal imaging FLIR tools software. The temperature scale was kept constant for tongue thermograms.  Figure 1 depicts the template of the tongue in which region of interest (ROI) is fixed on the tongue. The centre region of the tongue indicates the spleen and stomach organ of the human body [46]. So, the ROI has been fixed at the centre of the tongue region to classify DM.

**Figure 2:**
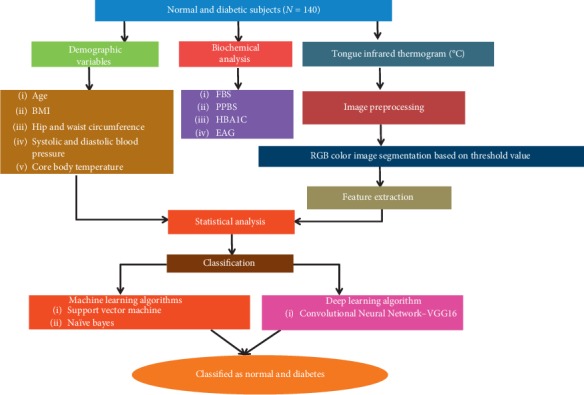
The proposed study design for the classification of type II DM.

The temperature was measured using various built-in tools such as spot detection, line, area, ellipse, rectangle, and so forth, available in FLIR tools software. Among all these tools, the rainbow palette and area tool was used to determine the ROI of the tongue thermograms. The shape of the ROI used was the rectangle area tool and the size of the area fixed in thermal imaging for measurement was 10 × 12 mm.  Figure 2 depicts the illustration of overall study design and the workflow of the proposed image processing technique in the diagnosis of type II DM.

The tongue thermogram was processed using Matlab R2014a software. The summary of an algorithm was given as follows:

  Step 1: tongue thermogram was considered as an input image
  Step 2: the images were resized into 256 × 256 pixels and preprocessed using Gaussian filter.
  Step 3: based on the preprocessed image histogram, minimum and maximum threshold values have been defined for three channels (red, green, and blue) and were segmented into red, green, and blue components.
  Step 4: the segmented red component (hot spot) region was converted into grayscale image and the constant ROI has been fixed in the centre of the tongue.
  Step 5: the statistical features were extracted from the desired ROI using the Gray level co-occurrence matrix (GLCM) algorithm.

### 2.4. RGB Based Color Histogram Image Segmentation

Image segmentation is a method of labelling every pixel in an image. The importance of image segmentation is to group each pixel in the related regions. The similar regions in the image can be perceived and clustered by using some properties like gray levels, color, texture, and intensity to obtain meaningful information [47]. Image segmentation can be performed based on discontinuity or similarity property of the intensity values. The thresholding method is the similarity property for partitioning the images. Thresholding is a technique which partitions the image into foreground and background based on the brightness regions in the image. The gray image thresholding is as follows in equation (1):

(1) $$\begin{array}{c} g\left(m,n\right)=\left\{\begin{array}{c} \text{if }f\left(m,n\right),<T\text{ then }0,\\ \text{else,}\\ f\left(m,n\right)\geq T\text{ then }1, \end{array}\right. \end{array}$$

where *T* is the single threshold value, *f* (*m*, *n*) is the input gray level image, and *g* (*m*, *n*) is the output threshold image. In the color image segmentation, each and every pixel is categorized by RGB channels. The threshold value of each channel should be defined for three RGB components such that the foreground pixel values fall in the selected range of RGB intensities of the image [48]. The RGB based color histogram image segmentation is as follows in equation (2):

(2) $$\begin{array}{c} G_{r,g,b}\left(m,n\right)=\left\{\begin{array}{c} \text{if }r\left(m,n\right),<T\text{ then }0,\\ \text{else,}\\ r\left(m,n\right)\geq T\text{ then }1,\\ \text{if }g\left(m,n\right),<T\text{ then }0,\\ \text{else,}\\ g\left(m,n\right)\geq T\text{ then }1,\\ \text{if }b\left(m,n\right),<T\text{ then }0,\\ \text{else,}\\ b\left(m,n\right)\geq T\text{ then }1, \end{array}\right. \end{array}$$

where *G*_*r,g,b*_ (*m*, *n*) is the threshold output image of red, green, and blue components, *T* is the threshold value, and input RGB components are *r* (*m*, *n*), *g* (*m*, *n*), and *b* (*m*, *n*) of the image. The color image segmentation in the proposed study has been performed based on the RGB based color histogram thresholding technique. It calculates the RGB histogram of the image with three channels. The range of minimum and maximum threshold values for RGB components with three RGB channels is defined and tabulated in Table 2. Based on this defined minimum and maximum threshold values, the tongue thermogram has been segmented into red, green, and blue components separately.

### 2.5. Statistical Feature Extraction

The RGB components were segmented from the preprocessed tongue thermogram. The segmented red component was converted into grayscale output image. The feature extraction technique was applied over the grayscale image. The desired ROI was fixed at the middle of the grayscale tongue image to extract the intensity and statistical features. The GLCM algorithm was implemented in the grayscale tongue image to extract the features such as contrast, correlation, energy, homogeneity, mean, entropy, standard deviation, skewness, variance, and kurtosis. The definition of the GLCM parameters was given as follows:

  Contrast: it is a measure of the intensity contrast between a pixel and its neighbour over the whole tongue image and it is calculated by the following equation:

(3) $$\begin{array}{c} \overset{N-1}{\underset{i,j=0}{\sum }}P\left(i,j\right){\left(i-j\right)}^{2}. \end{array}$$

  Correlation: it is the measure of how correlated a pixel is to its neighbor over the whole tongue image and it is defined in the following equation:

(4) $$\begin{array}{c} \overset{N-1}{\underset{i,j=0}{\sum }}\frac{P\left(i,j\right)\left(i-\mu \right)\left(j-\mu \right)}{\sigma^{2}}. \end{array}$$

  Energy: it measures the textural uniformity. The energy value is highest when all the values in the co-occurrence matrix (GLCM) are all equal. It can be computed using the following equation:

(5) $$\begin{array}{c} \overset{N-1}{\underset{i,j=0}{\sum }}P{\left(i,j\right)}^{2}. \end{array}$$

  Homogeneity: it computes the closeness of the conveyance of segments in the GLCM to the GLCM corner to corner. It is represented mathematically as follows:

(6) $$\begin{array}{c} \overset{N-1}{\underset{i,j=0}{\sum }}\frac{P\left(i,j\right)}{\left(1+{\left(i-j\right)}^{2}\right)}. \end{array}$$

  Mean: it measures the average gray level of every district in a tongue image. The mean can be calculated using the following equation:

(7) $$\begin{array}{c} \overset{N-1}{\underset{i,j}{\sum }}iP\left(i,j\right). \end{array}$$

  Standard Deviation: it is a proportion of variation from the mean esteem and can be calculated using the following equation:

(8) $$\begin{array}{c} {\left(\overset{N-1}{\underset{i,j=0}{\sum }}{\left(i-\mu \right)}^{2}P\left(i,j\right)\right)}^{1/2}. \end{array}$$

  Skewness: it calculates the asymmetry of the data around the sample mean and it is defined as follows:

(9) $$\begin{array}{c} \sum \frac{{\left(Xi-µ\right)}^{3}}{n\sigma^{3}}. \end{array}$$

  Variance: it calculates the gray level vacillations from the mean gray level dimension value. It can be calculated as follows:

(10) $$\begin{array}{c} \overset{N-1}{\underset{i,j=0}{\sum }}P\left(i,j\right){\left(i-\mu \right)}^{2}. \end{array}$$

  Kurtosis: it measures about the outlier prone distribution and describes the shape of the tail of histogram and is mathematically represented in the following equation:

(11) $$\begin{array}{c} \sum \frac{{\left(Xi-µ\right)}^{4}}{n\sigma^{4}}. \end{array}$$

  Entropy: it is a proportion of randomness that can be utilized to describe the texture of a tongue image and can be calculated by using the following equation:

(12) $$\begin{array}{c} \overset{N-1}{\underset{i,j=0}{\sum }}-\ln\left(P\left(i,j\right)\right). \end{array}$$

where *P* (*i*, *j*) = Elements *i* and *j* of the gray levels in a grayscale tongue image, *N* = No. of gray levels in the grayscale tongue image, *n* = sample size, *σ*^2^ = Variance, *µ* = Mean, and *σ* = Standard Deviation.

### 2.6. Machine Learning Algorithms

To discriminate the normal and type II DM and obtain the best classification accuracy, the machine learning algorithms such as Support Vector Machine (SVM), Naïve Bayes (NB), and deep learning algorithm such as Convolutional Neural Network (CNN) VGG 16 net were used in our proposed study. The input parameters such as anthropometrical body circumferences, blood pressure, measured temperature at the tongue region, and extracted GLCM features at desired ROI were fed into SVM and NB algorithms using Matlab programming environment. The input images were fed directly into CNN using Python programming environment. The tenfold cross validation was performed to obtain better classification accuracies.

### 2.7. SVM Classifier

The SVM algorithm is one of the outstanding supervised machine learning algorithms dependent on factual learning hypothesis. It uses an ideal linear separating hyperplane to partition the two sets of information in the feature space [49]. The given training sets for two classes are (*xm*, *ym*), (*xk*, *yk*), (*xn*, *yn*), where *xi* = 2D feature space and *yi* = {−1, +1} class labels with *i* = 1,…, *n*. The SVM classifier fabricates the ideal separable hyperplanes based on the kernel function (*K*). Training the SVM in the linear kernel *K* (*x*, *y*) = <*x*, *y*> is much faster than the other kernels. The feature vectors of the image lie on hyperplane's one side that are fitted into the −1 class and the remaining feature vectors belong to the +1 class.

### 2.8. NB Classifier

The NB algorithm is a probabilistic grouping algorithm dependent on the Bayes hypothesis and is particularly fit when the dimensionality of the free spaces (i.e., number of inputs) is high [50]. From a given set of independent variables, *X* = {*x*1, *x*2, *x*3,…, *xn*}, where *X* is a predictor. The condition probability is *P* (*C*_*k*_, *X*) = *P* (*C*_*k*_|*X*).*P* (*X*) = *P* (*X*|*C*_*k*_).*P* (*C*_*k*_) for each *k* class where *k* = 1, 2. Using the Bayes theorem to construct the conditional probability, *P* (*C*_*k*_|*X*) = (*P* (*X*|*C*_*k*_).*P* (*C*_*k*_))/*P* (*X*).

### 2.9. CNN Classifier

**Figure 3:**
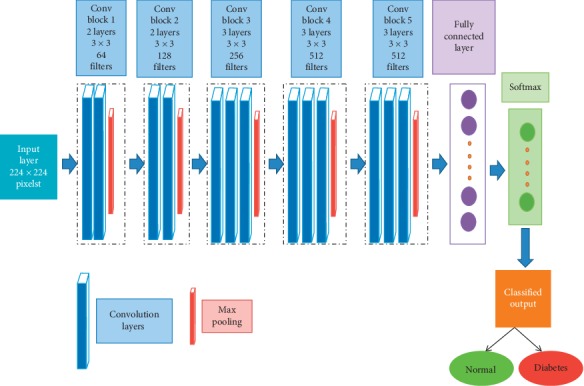
VGG16 Convolutional Neural Network classifier architecture.

Convolutional Neural Network is a class of deep learning architectures which has turned out to be one of the dominant models in the world of computer vision. CNN is a mathematical construct which is basically composed of stacking of multiple building blocks with three layers such as convolution layers, pooling layers, and fully connected layers. CNN can automatically extract and adapt to learn the spatial features from the image. A convolutional layer plays a vital role in CNN architecture with a stack of mathematical linear and nonlinear operations to perform the feature extraction. The pooling layer performs the downsampling operation and decreases the number of learnable parameters. The extracted and pooled features from convolution and pooling layers are mapped in the fully connected layers to the final output. Currently there are many pretrained deep learning CNN models such as Alex Net [51], Res Net [52], Dense Net [53], and VGG net [54] which are available for the classification. As per the literature, the VGG net architecture is the most commonly used deep learning CNN for medical image classification. Hence, it has been used in our proposed study to discriminate the normal and diabetic subjects.  Figure 3 portrays the pretrained VGG16 net CNN architecture. The transfer learning approach was used in VGG16 net architecture with five convolutional blocks and thirteen convolutional layers. One of the methods used in our proposed study to reduce the overfit in the model is the data augmentation method which modifies the training data through random transformations. The data augmentation methods such as shearing, zooming, and rotational, horizontal, and translational flipping were used in VGG16 net CNN architecture. The max pooling (2D) method was used for pooling operation in the pooling layer. Stochastic Descent Gradient (SDG) was used as an optimization algorithm in the VGG16 net that updates the learning parameters iteratively with a 0.01 learning rate. The Rectified Linear Unit (ReLu) activation function is used in the fully connected layer of VGG16 net and softmax function which normalizes the output values and target class probabilities ranges 0 or 1.

### 2.10. Receiver Operating Characteristic Curve (ROC) and Performance Assessment Metrics

The ROC curve is a graphical plot between true positive rate and false positive rate which elucidates the performance of different classifiers. The diagnostic accuracy of the test can be interpreted from the area under the ROC curve [55].

(i) Sensitivity (%) is the proportion of true prediction of type II DM.
    Sensitivity = TP/(TP + FN)
(ii) Specificity (%) is the proportion of true prediction of normal.
    Specificity = TN/(TN + FP)
(iii) Accuracy (%) is the proportion of true prediction of the present study.
    Accuracy = (TP + TN)/(TP + TN + FP + FN)
(iv) Positive Predictive Value (%) = TP/(TP + FP)
(v) Negative Predictive Value (%) = TN/(TN + FN)
True Positive (TP): the subjects are correctly identified as type II DM.
False Positive (FP): the subjects are incorrectly identified as type II DM.
True Negative (TN): the subjects are correctly identified as normal.
False Negative (FN): the subjects are incorrectly identified as normal.

### 2.11. Statistical Analyses

All the statistical studies were performed using SPSS (IBM) software package version 21.0, Chicago, IL, USA. All the data were expressed as mean ± standard deviation (S.D). Student's *t*-test was computed for all the parameters such as anthropometric, biochemical, measured tongue temperature, and extracted features for the total studied population. The Shapiro–Wilk test was performed for data normalization. The Kruskal–Wallis was a rank based nonparametric test performed between the groups to determine the statistically significant difference. The Pearson correlation was studied to examine the association between the different variables.

## 3. Results and Discussion

The tongue thermal pattern distribution may fluctuate due to many myriad factors such as fever, arthritis, and anaemia, and pregnant women can have hormonal changes, renal problems, and uncontrolled blood pressure which lead to cardiovascular complications. The certain factors observed to lower the diagnostic efficiency of tongue thermograms to classify the type II DM in the prior stage were excluded from the proposed study based on the questionnaires obtained from the total subjects.

Table 3 indexed the baseline characteristics (mean ± SD) of all subjects grouped into normal and type II DM. It was observed that only age (years) in anthropometrical parameter and waist circumference (cm) in body circumference variable shows a significant difference between the normal and type II DM subjects. The blood pressure parameters had not produced any significant differences between the normal and diabetic groups. The biochemical parameters such as HbA_1c_ (%), glucose (mg/dl), and EAG (mg/dl) show highly statistically significant difference between the groups (*p* < 0.01).

The temperature measured at ROI shows statistically significant differences in the tongue region between the groups. The statistical features extracted from tongue thermograms such as contrast, energy, mean, standard deviation, entropy, skewness, variance, and kurtosis show a significant difference between normal and DM group (*p* < 0.01), whereas the other features like correlation and homogeneity did not show the statistical difference between the groups.

**Figure 4:**
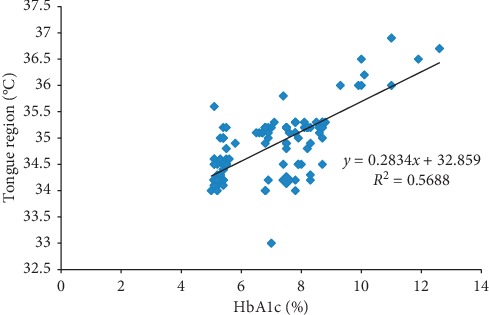
Scatter plot between HbA_1c_ (%) and thermal distribution in the tongue region (°C).

Figure 4 represents the scatter plot showing the statistical correlation (*r*^2^ = 0.5688) between the HbA_1c_ (%) and thermal distribution in the tongue region (°C).

**Figure 5:**
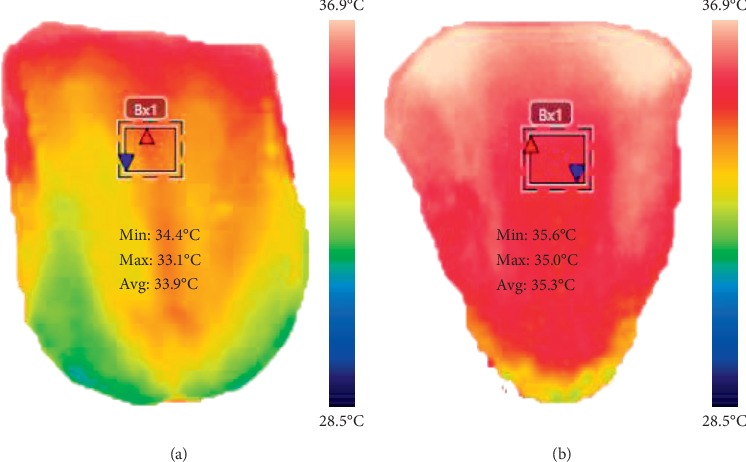
Measured sample tongue thermograms. (a) Sample normal subject. (b) Sample diabetic subject.

Figure 5 represents the sample measured tongue thermograms. Figures 5(a) and 5(b) represent the measured thermal distribution of the samplenormal and diabetic tongue thermograms acquired from an infrared camera. The temperature scale was kept constant for normal and diabetic tongue thermograms. The red and blue arrows in the ROI box indicate the higher and lower temperatures in the tongue region.

**Figure 6:**
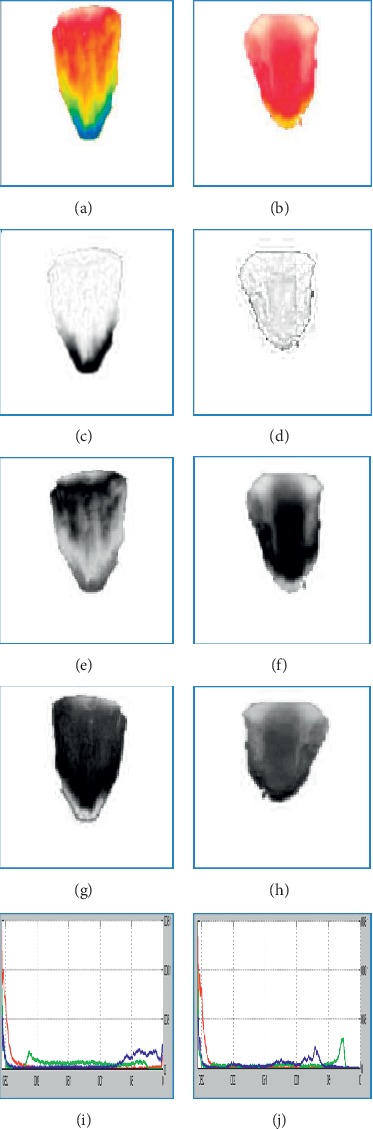
RGB color channels and histogram of the tongue thermogram. (a, b) Sample normal and diabetic tongue thermograms. (c, e, g) Red, green, and blue channels of the sample normal tongue thermogram. (d, f, h) Red, green, and blue channels of the sample diabetic tongue thermogram. (i, j) RGB color histogram of the sample normal and diabetic subjects.

Figure 6 portrays the normal and diabetic RGB channels and histograms of tongue thermogram. Figures 6(a) and 6(b) depict the normal and diabetic tongue thermograms. Figures 6(c), 6(e), and 6(g) portray the red, green, and blue channels of normal tongue thermogram. Similarly, Figures 6(d), 6(f), and 6(h) represent the diabetic tongue thermogram. Figures 6(i) and 6(j) represent the RGB histogram of normal and diabetic subjects.

**Figure 7:**
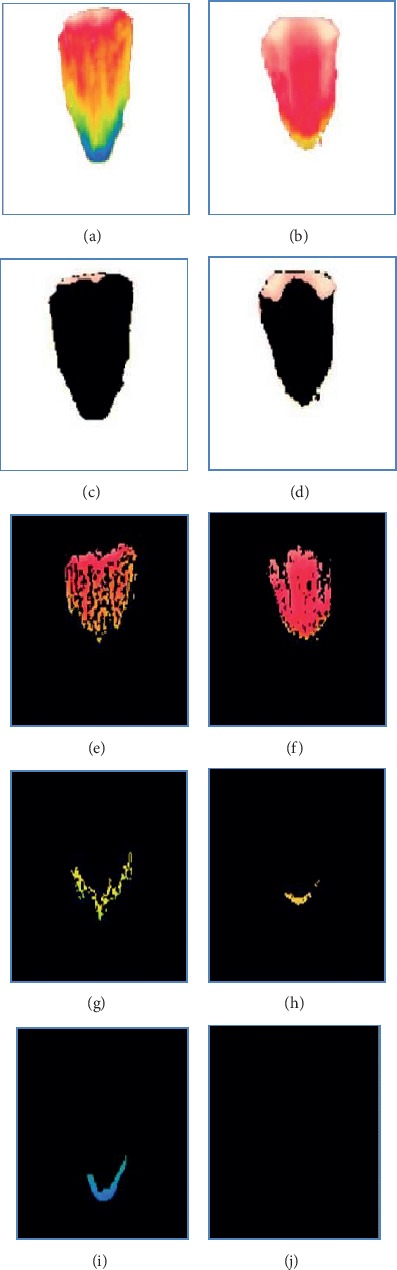
Segmented RGB color components of tongue thermogram. (a, b) Sample normal and diabetic tongue thermograms. (c, d, e, f) Segmented white and red color in the sample normal and diabetic tongue thermograms. (g, h, i, j) Segmented green and blue color from the sample normal and diabetic tongue thermograms.

Figure 7 represents the segmented RGB of normal and diabetic tongue thermograms. Figures 7(a) and 7(b) are the tongue thermograms of normal and diabetic subjects. Figures 7(c)–7(f) represent the segmented white and red components from normal and diabetic tongue thermograms. Figures 7(g)–7(j) represent the segmented green and blue components from the normal and diabetic tongue thermograms. The lack of blue component was observed in Figure 7(j) which indicates the absence of cold spot in the diabetic tongue. From these segmented results, the red component is a hot spot which was found to be lower in the normal subjects compared to diabetic individuals. Also, the thermal patterns were found to be limited in the centre region of the diabetic tongue whereas the thermal patterns were found to be in the overall region for normal tongue.

Table 4 represents the Pearson correlation matrix for diabetic subjects (*n* = 70) for all the parameters. The diastolic blood pressure (DBP) of the diabetic group was positively correlated with HbA_1c_ (*r* = 0.246, *p* < 0.05). The HbA_1c_ was positively correlated with FBS (*r* = 0.647, *p* < 0.01), PPBS (*r* = 0.711, *p* < 0.01), and EAG (*r* = 0.555, *p* < 0.01) for the diabetic population, as it was obvious that increased FBS, PPBS, EAG levels lead to increased HbA_1c_ level. The HbA_1c_ was positively correlated with the measured temperature of the tongue (*r* = 0.662, *p* < 0.01).

Table 5 represents the confusion matrices of (a) SVM, (b) NB, and (c) CNN classifiers. The sensitivity, specificity, and accuracy were found from the confusion matrix.  Table 6 denotes the performance of three different classifiers for the classification of normal and diabetic groups. Among the three different classifiers, it was observed that the CNN classifier achieved a better accuracy rate as 94.28% than the NB (89.28%) and the SVM (92.85%) classifiers.

**Figure 8:**
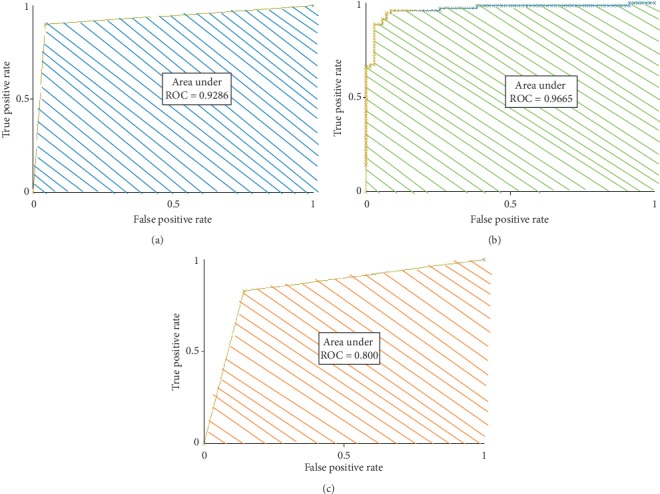
Receiver operating characteristic (ROC) curves of various classifiers. (a) ROC of the SVM classifier. (b) ROC of the NB classifier. (c) ROC of the CNN classifier.

**Figure 9:**
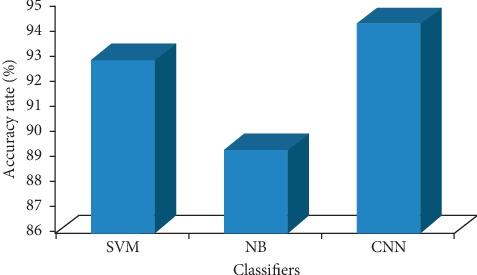
Accuracy rate bar diagram of different classifiers.

**Figure 10:**
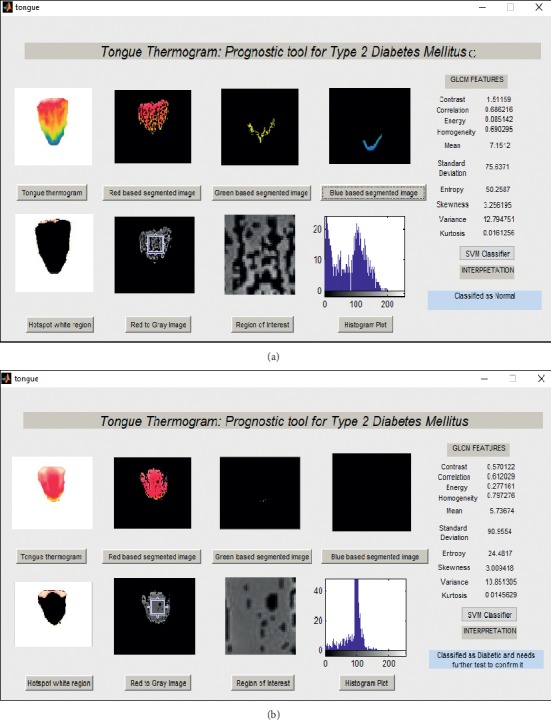
Computer aided diagnostic (CAD) system for the classification of type II diabetes mellitus. (a) Sample normal subject. (b) Sample diabetic subject.

Figures 8(a)–8(c) represent the area under the ROC curve of support vector machine (SVM), Naïve Bayes (NB), and Convolutional Neural Network (CNN) classifiers.  Figure 9 shows the accuracy rate bar diagram of different classifiers. Figures 10(a) and 10(b) show the computer aided diagnostic system for tongue thermogram for classifying the sample normal and diabetic subject using SVM classifier based on the measured mean tongue temperature and extracted statistical features at the desired region of interest in tongue thermogram.

The present study analysed the thermal distributions in the tongue thermogram between the normal and type II DM. The RGB color based histogram thresholding method is used for segmentation of tongue thermogram. The normal subjects were distinguished from the diabetic with a better accuracy rate using CNN classifier as a novel approach for classification of type II DM. The GLCM algorithm was used to extract the statistical features at the constant ROI from the red component which has been converted into grayscale tongue image. The percentage difference of measured average temperature of tongue region between normal and diabetic groups was found to be 2.35%. The temperature measured at constant ROI of the tongue was correlated with HbA_1c_ biochemical parameter. The hot region (red component) was spotted to be higher in the centre region of the diabetic tongue than the normal. The biochemical variables, measured temperature at tongue region and extracted features, were found to be statistically significant. Further, the normal and diabetic subjects were classified using SVM, NB, and CNN classifiers. Finally, the CAD system was developed for the classification of type II diabetic from tongue thermogram using the SVM classifier. To the best of our knowledge and as per the review of literature, the studies related to the tongue thermograms were limited. Also, the classification of type II DM based on tongue thermogram using deep learning methods was not initiated till today.

The poor glycaemic control in diabetic subjects leads to the cardiovascular problems; therefore tight glycaemic control (HbA_1c_ of <7%) recommended for the prevention of the onset of disease was discussed by Moodahadu et al. [56]. The study computed by Conget and Gimenez [57] elucidated the relationship between the glucose control and cardiovascular disease. The endothelial cells assume a fundamental role in maintaining the cardiovascular homeostatic balance if these cells lose their physiological properties which leads to the vasodilation and development of atherosclerosis. In spite of good glucose control, the endothelial cell levels were found to be higher in the diabetic subjects. The poor or fluctuating glycaemic control in the subjects lead to endothelial dysfunction and oxidative stress which was briefed by Avogaro et al. [58] and Ceriello et al. [59]. In our study, the positive correlation (*r* = 0.246, *p* < 0.05) was observed between the diastolic blood pressure and HbA_1c_ in the studied diabetic group; this might lead to the cardiovascular complications for diabetic subjects in the future.

According to the literature, the tongue thermograms were observed with decreased temperature for various diseases. The tongue thermograms of the anaemic subjects were observed to have a lower temperature (34.14°C) by the study conducted by Xie and Zhang [38]. And Baek et al. have found decline in tongue temperature (32.70°C) for cold and heat patterns of the women subjects with gynaecological problems [41]. Whereas in the case of type I diabetic tongue thermograms the temperature was found to be higher (35.94°C) than other diseases observed by Selvarani and Suresh [60]. Similarly in our study the type II diabetic tongue thermogram which falls in the temperature range between 35.23°C and 35.84°C at the desired region of interest could be one of the parameters for the classification of type II diabetes mellitus.

Sreebny et al. observed a lower salivary flow rate (<0.1 to <0.7 ml/min) in diabetic compared with healthy subjects [61]. Lin et al. speculated that the impaired salivary rate leads to noninsulin dependent DM with xerostomia conditions [62]. An increased mean surface temperature at the tongue region was observed in our diabetic group compared with normal group. The variations in the glucose levels are almost influenced by the secretion of saliva. The upsurge in measured tongue temperature was observed in the diabetic group because of the lower secretion of saliva and xerostomia conditions.

Chiu in his study predicted that complications in the human stomach region can be diagnosed by the tongue color appearance and texture. They extracted statistical features such as angular second moment, variance, contrast, and entropy which are obtained in the tongue region of the random subjects. The grimy coating was detected in four subregions such as the liver, stomach, gall bladder, and heart using spatial gray tone dependency matrices (SGTDM) [63]. In our study, the extracted features like contrast, energy, mean, standard deviation, entropy, skewness, variance, and kurtosis were found to be statistically significant in both normal and diabetic groups. Whereas homogeneity and contrast features were not found to be insignificant between the groups. This insignificance might be due to the thicker white coating in the middle region of the diabetic tongue.

The classification of normal and diabetic neuropathy using plantar thermograms by interdigital isothermal technique was observed to have 81.3% and 46.2% as sensitivity and specificity [64]. The sensitivity of foot thermogram for screening the osteomyelitis was found to be 60% and observed increased skin surface temperature distribution in periwound ankle and knee areas of diabetic foot [65]. Zhang et al. extracted the color and texture features from the digital tongue images from diabetic and nondiabetic subjects to develop the diagnostic technique for DM. They observed the SVM classifier has comparatively achieved higher accuracy rate (79.72%) than k-NN (78.77%), NB (75.94%), and backpropagation neural network (75.00%) [66].

In our study, the sensitivity was found to be 90% in SVM, 95.71% in NB, and 92.85% in CNN for the classification of type II DM. It was observed from the review of literature that many methods rely on the handcrafted features and traditional classifiers such as SVM, NB, k-NN, random forest, and fuzzy sets for diagnosis and prognosis of the diseases. Deep learning architectures were found to be an evident tool in the world of machine vision especially CNN classifiers which paved the way for more accurate results in medical imaging [67]. We obtained competitive results compared to the traditional classifiers and achieved 94.28% as accuracy rate in CNN (VGG16 net) classifier (deep learning) for distinguishing the normal from the diabetic subjects using tongue thermograms. The limitation of the proposed study was needed to perform validation of the data for larger number of sample size. Inevitably, the conventional tongue evaluation does have its impediments inferable to both physician's ability and environmental setting.

## 4. Conclusions

In this present study, we have observed the measured tongue surface temperature was correlated significantly with the standard biochemical parameters. The measured tongue surface temperature of the diabetic group was found to be greater than the normal group. The statistical correlation between the HbA_1c_ and thermal distribution in the tongue region was found to be *r*^2^ = 0.5688. The baseline variables, tongue temperature parameters, and extracted features were fed as input attributes to the machine learning algorithms (SVM, NB), and input images were directly fed into the deep learning algorithm (CNN). The performances of the machine and deep learning algorithms were compared, and it was found that CNN classifier have outperformed the other classifiers with an overall accuracy rate of 94.28% whereas SVM classifier has obtained 92.85% and NB classifier has attained 89.28% for categorising the normal and type II DM. And, finally a computer aided diagnostic system was developed for the classification of type II DM. Diabetes is one of the life threatening disorders so the early assessment is most important to control the prevalence of the disease. So, the tongue thermogram assessment may conceivably fill in as a prompt, reasonable, and noninvasive approach for preliminary screening of type II DM.

## Figures and Tables

**Table 1:** Total population classified into two groups based on a diagnostic criterion.

Parameters	Group I: normal subjects (*n* = 70)	Group II: type II diabetes mellitus subjects (*n* = 70)
HbA_1c_ (%)	≤6.4	≥6.5
Male/female ratio	1 : 2	1 : 2
Age (years), mean (±SD)	43.88 (±10.71)	47.38 (±7.42)

**Table 2:** Tongue thermogram: RGB components threshold values.

Color components	Red channel		Green channel		Blue channel	
	Threshold values		Threshold values		Threshold values	
	Minimum	Maximum	Minimum	Maximum	Minimum	Maximum
Red	222	252	0	176	0	122
Green	8	255	165	255	6	96
Blue	0	28	0	161	0	255

**Table 3:** Baseline characteristics of total population (*N* = 140).

Parameters & GLCM features	Normal (*N* = 70)	Diabetes (*N* = 70)	*p* value
Age (years)	43.88 ± 10.71	47.38 ± 7.42	0.02 (s)
Height (cm)	160.90 ± 6.95	159.27 ± 10.17	0.27 (ns)
Weight (kg)	63.48 ± 13.54	66.06 ± 11.76	0.23 (ns)
BMI (Kg/m^2^)	24.51 ± 4.98	26.13 ± 5.45	0.06 (ns)
Onset of diabetes duration (years)	—	8.78 ± 6.26	—
Spo_2_ (%)	97.9 ± 0.81	98.1 ± 0.68	0.11 (ns)
Core body temperature (°C)	36.87 ± 0.30	36.93 ± 0.42	0.33 (ns)
Waist circumference (cm)	79.57 ± 12.44	84.51 ± 14.89	0.01 (s)
Hip circumference (cm)	87.22 ± 11.69	91.75 ± 17.38	0.07 (ns)
Systolic pressure (mmHg)	122 ± 15.18	122.85 ± 18.42	0.07 (ns)
Diastolic pressure (mmHg)	76.42 ± 9.33	76.14 ± 9.82	0.86 (ns)
HbA_1c_ (%)	5.27 ± 0.15	8.58 ± 2.30	0.0001 (s)
FBS (mg/dL)	94.31 ± 10.04	148.67 ± 58.72	0.0001 (s)
PPBS (mg/dL)	116.02 ± 15.15	225.32 ± 98.73	0.0001 (s)
EAG (mg/dL)	119.77 ± 7.21	191.1 ± 66.57	0.0001 (s)
Tongue surface temperature (°C) ROI	34.62 ± 0.77	35.23 ± 0.61	0.0001 (s)
Contrast	0.86 ± 0.40	0.74 ± 0.23	0.0412 (s)
Correlation	0.78 ± 0.04	0.76 ± 0.08	0.1269 (ns)
Energy	0.27 ± 0.17	0.19 ± 0.04	0.0001 (s)
Homogeneity	0.80 ± 0.07	0.79 ± 0.02	0.7066 (ns)
Mean	6.17 ± 0.90	6.51 ± 0.41	0.0047 (s)
Standard deviation	47.75 ± 18.42	79.91 ± 11.01	0.0001 (s)
Entropy	45.88 ± 5.12	41.63 ± 10.55	0.0029 (s)
Skewness	3.78 ± 0.88	2.82 ± 0.35	0.0001 (s)
Variance	18.37 ± 8.98	10.38 ± 1.76	0.0001 (s)
Kurtosis	0.01 ± 0.006	0.02 ± 0.008	0.0018 (s)

s: significant; ns: nonsignificant.

**Table 4:** Pearson correlation of studied type II diabetes mellitus subjects (*n* = 70).

	Age (years)	Hip cir (cm)	SBP (mmHg)	DBP (mmHg)	HbA_1c_ (%)	FBS (mg/dl)	PPBS (mg/dl)	EAG (mg/dl)	TST (°C)
Age (years)	1								
Hip cir (cm)	0.458^*∗∗*^	1							
Waist cir (cm)	0.305^*∗*^	0.780^*∗∗*^	1						
DBP (mmHg)	0.047	−0.108	0.718^*∗∗*^	1					
HbA_1c_ (%)	−0.082	0.076	0.235	0.246^*∗*^	1				
FBS (mg/dl)	−0.039	0.047	0.082	0.115	0.647^*∗∗*^	1			
PPBS (mg/dl)	0.007	0.099	0.093	0.071	0.711^*∗∗*^	0.653^*∗∗*^	1		
EAG (mg/dl)	−0.033	0.003	0.161	0.198	0.555^*∗∗*^	0.834^*∗∗*^	0.552^*∗∗*^	1	
TST (°C)	−0.009	−0.036	0.19	0.276^*∗*^	0.662^*∗∗*^	0.435^*∗∗*^	0.471^*∗∗*^	0.270^*∗*^	1

^*∗∗*^correlation is significant at 0.01 level, ^*∗*^correlation is significant at 0.05 level. Hip cir: hip circumference (cm), waist cir: waist circumference (cm), SBP: systolic blood pressure, DBP: diastolic blood pressure, HbA_1c_: glycated haemoglobin, FBS: fasting blood sugar, PPBS: postprandial blood sugar, EAG: estimated average glucose, TST: tongue surface temperature at region of interest.

**Table 5:** Confusion matrices of all the classifiers.

(a) Support vector machine (SVM)	Normal (predicted)	Diabetes mellitus (predicted)
Normal (actual)	67 (TN)	3 (FP)
Diabetes mellitus (actual)	7 (FN)	63 (TP)

(b) Naïve Bayes (NB)	Normal (predicted)	Diabetes mellitus (predicted)
Normal (actual)	58 (TN)	12 (FP)
Diabetes mellitus (actual)	3 (FN)	67 (TP)

(c) Convolutional Neural Network (CNN)	Normal (predicted)	Diabetes mellitus (predicted)
Normal (actual)	67 (TN)	3 (FP)
Diabetes mellitus (actual)	5 (FN)	65 (TP)

TP: true positive, FP: false positive, TN: true negative, FN: false negative, PPV: positive predictive value, NPV: negative predictive value.

**Table 6:** Comparison of classification performance of SVM, NB, and CNN machine learning algorithms.

Classifiers	AUC	Sensitivity (%)	Specificity (%)	PPV (%)	NPV (%)	Accuracy (%)
Support vector machine (SVM)	0.92	90	95.71	91.30	90.54	92.85
Naïve Bayes (NB)	0.96	95.71	82.85	84.81	95.08	89.28
Convolutional Neural Network (CNN-VGG16 net)	0.80	92.85	95.71	95.58	93.05	94.28

AUC: area under the curve (ROC), PPV: positive predictive value, NPV: negative predictive value.

## Data Availability

The data used to support the findings of this study have not been made available because it is restricted in order to protect the patient's privacy.
